# Maintaining Patency of Vascular Access for Haemodialysis

**DOI:** 10.1007/s13239-017-0320-3

**Published:** 2017-07-18

**Authors:** Nicholas Inston, J. Al Shakarchi, A. Khawaja, R. Jones

**Affiliations:** 1 Department of Renal Surgery, University Hospital Birmingham, Birmingham, UK; 2 Department of Radiology, University Hospital Birmingham, Birmingham, UK

**Keywords:** Thrombosis, Maturation, Vascular access, Patency, Arteriovenous fistula

## Abstract

All types of vascular access, a necessity for haemodialysis, are prone to thrombosis and if untreated this results in failure. Thrombosis results from the combination of impaired blood flow, endothelial and vessel wall injury and a propensity towards pro-coagulative states, either intrinsic or aggravated by dialysis or dehydration. The treatment of access thrombosis relies on removal of the clot (thrombectomy) and treatment of the underlying problem. In most cases this is stenosis secondary to neointimal hyperplasia which can occur early (failure to mature) or later. Pharmacological approaches have largely been shown to be ineffective at prevention of thrombosis. The mainstay of preventing access failure may be in surveillance and detecting stenosis prior to occlusion although the optimal protocol to achieve this remains undefined. Management of thrombosed access is *via* either surgical and radiological approaches. Multiple techniques and devices are available for thrombectomy and the choice is usually based on local expertise and availability rather than evidence as few trials have been performed to allow robust comparisons. This paper outlines the basis of access thrombosis and discusses the currently available techniques for treatment.

## Introduction

Haemodialysis vascular access (VA), whether an autologous arteriovenous fistula (AVF) or a prosthetic arteriovenous graft (AVG), is prone to thrombosis which, if untreated, results in failure. Prior to thrombosis occurring, fistulas may function normally although those with poor flows or high pressures due to underlying stenosis are at high risk.^15^ Following formation a proportion of AVFs fail to mature (FTM) and are unsuitable for dialysis.^1^,^4^ In many studies FTM is termed thrombosis and should be more accurately defined.^24^

The pathogenesis of thrombosis results from activation of the clotting cascade and the factors responsible were first described by Virchow in 1888:^17^

1. Stasis of blood.
2. Blood vessel wall injury.
3. Change in constituents of the blood.

When these are applied to vascular access it is clear why the occurrence of thrombosis is high and is the usual eventual fate of both AVFs and AVGs (Table 1). During surgical creation of VA, there is stasis due to vascular clamping. There is vessel wall injury secondary to surgical trauma, clamps and sutures. The constituents of the blood may be altered with higher blood viscosity and dehydration from dialysis and preoperative fasting. Some patients may have pro-coagulative tendencies. Chronic kidney disease in itself has been reported to represent a strong and independent risk factor for both spontaneous venous and arterial (post-vascular injury) thrombosis.^17^ As a result primary failure rates are high. Even with successful post-operative flow the response of the vessel wall to abnormal flow patterns may result in a neointimal hyperplastic (NIH) response with progressive stenosis and resistance to flow with localised stasis.^8^,^14^ This process affects between 20 and 60% of AVFs and AVGs.^3^,^13^,^24^

**Table 1:** Factors involved in the pathogenesis of dialysis vascular access thrombosis as described by Virchow’s triad.

	Stasis of blood	Wall injury	Content of blood
At surgery	Clamping of vessels Vessel spasm	Surgical trauma Clamps Sutures uraemia	Dehydration Post dialysis haemoconcentration Erythropoietin
Maturation	Neointimal hyperplasia Vein fibrosis due to venepuncture	Pathological flow e.g. turbulence Uraemia Diabetes	Procoagulant states
Dialysis	Pressure on needle points Stenosis due to neointimal hyperplasia	Repeated needling Aberrant flow Local paracrine factors	Dehydration Hypotension

Even in established VA the continued effects of aberrant vascular flow cause stenosis due to NIH and in association with repeated needling along with problems such as dehydration and hypotension predispose to thrombosis and failure.

Clinically many dialysis established patients will be the ones to make the diagnosis of VA thrombosis as they no longer feel a thrill within the access. If not, the dialysis nurses will be unable to use the access at their next dialysis session. Final confirmation with sonography may be required. The urgency of thrombectomy is higher in AVFs due to the risk of further endothelial injury by continued thrombus contact. Theoretically AVGs can be successfully salvaged up to 2 weeks or beyond, although the requirement for dialysis would favour early intervention. Surgical and interventional radiological techniques are available and may be used in combination.^22^

## Surgical Thrombectomy

Surgical thrombectomy in its simplest form involves open surgical access to the fistula and clot expression by manual pressure. This is of course a crude technique with poor results and the introduction of the Fogarty balloon catheter in 1966 resulted in much improved outcomes.^6^ It is recommended that on-table angiography should be performed whenever possible to exclude any stenoses or residual thrombus. If an underlying cause of thrombosis is identified, this should be treated straight away to avoid recurrence of the thrombosis. Surgical thrombectomy of an AVF can be especially challenging compared to a graft. It is often difficult to completely remove thrombus adjacent to the anastomosis of the fistula, and pseudoaneurysms within the fistula can prevent passage of the thrombectomy catheter or complete removal of thrombus from the fistula.

Studies that have shown improved outcomes from surgery over radiology tend to include complete revisional surgery of the access rather than true salvage procedures. Improvements in interventional radiology (IR) techniques have made the percutaneous modality to be more favourable. Percutaneous salvage of thrombosed AVFs has been reported to be a highly efficient procedure as well as being a significantly lower healthcare cost than surgical intervention.^2^ A recently published meta-analysis reported that comparable results to surgery were achieved with endovascular techniques for occluded prosthetic grafts for dialysis access. However long-term data comparing the two groups was reported to be lacking.^7^ The best results for surgical thrombectomy compared to radiological intervention is found in arteriovenous fistula when the cause of the thrombosis can be due to anastomotic stenosis as proximal re-anastomosis can be carried out to treat the underlying cause at the time of surgery.^22^

## Radiological Interventions

Nowadays in most centres IR techniques are the first line due to their minimally invasive nature. There are a multitude of devices available for percutaneous intervention for thrombosis with emerging evidence supporting their application.^9^,^10^,^18^ Endovascular therapy employs a number of different techniques and these can be divided as follow:

1. Catheter Directed Thrombolysis—The instillation of a lytic agent such as tPA (tissue plasminogen activator). This techniques allows the clot to be ‘laced’ with the agent and relying on it to subsequently dissolve to restore patency.
2. Mechanical Thrombectomy—There are a number of mechanical devices available that all work on the principle of macerating the clot and either dissipating it within the circulatory system or subsequently removing it by suction. Some examples of these include employment of rotating baskets (Arrow Treratola device^®^, PTD Arrow—Reading, PA, USA, The Cleaner™ Rotational Thrombectomy System—Rex medical, Athens, TX, USA) and brushes (Cragg thrombolytic brush catheter, TBC; MicroTherapeutics, Irvine, Calif, USA).^5^,^18^
3. Pharmacomechanical—This combines mechanical thrombectomy with thrombolysis and has shown to be very effective. An example of a device that delivers this is the AngioJet Rheolytic system^®^ (Boston Scientific, MA, USA).^20^ Here a high pressure jet of saline solution (which can be mixed with thrombolytic agents) is injected by a pump through a catheter that is then moved over a wire across the thrombosed segment of the target vessels. The high pressure solution fragments the clot and a negative pressure is created ahead of the catheter. With the subsequent negative pressure, the fragmented clot is retrieved *via* the catheter in its exhaust port. Another recently available device, the EKOS MicroLysUS infusion catheter^®^, (EKOS Corporation, Bothell, WA, USA) is based on ultrasound enhanced thrombolysis. This device instils tPA slowly *via* a very narrow catheter left indwelling in the thrombosed segment. Adjuvant disruption of the clot is achieved with an ultrasound cavitation effect.^25^ This has been shown to be effective in various settings, various settings but little evidence is currently available for its application in vascular access thrombosis.^16^
4. Suction Thrombectomy: This is the removal of culprit thrombus *via* a large bore catheter placed percutaneously within the vessel and attached manually to a large bore syringe. This is particularly effective at treating relatively fresh thrombus.
5. Any combination of the above techniques can be applied.

Whichever modality is applied for thrombus removal there is always an underlying pathology, usually a mechanical obstruction causing the thrombosis, such as a venous stenosis. This needs to be treated at the same time as clot removal to restore flow in the access and prevent future re-thrombosis. Balloon angioplasty is usually effective but high elastic recoil forces in diseased areas often require a stent or stent graft to maintain and improve patency.^23^ Stents offer mechanical radial support in these circumstances but controversially themselves are also prone to re-narrowing due to intimal hyperplasia and subsequent thrombosis. Covered stents or stent grafts may avoid this phenomenon of in-stent NIH. However, these too are prone to its formation and subsequent stent graft edge stenosis.

## Conclusion

Vascular access thrombosis can be treated by both surgical and radiological thrombectomy. Through technological advancements, intervention radiology has become the first choice therapy. The benefits of interventional radiology extend beyond the minimally invasive approach by allowing visualisation and treatment of the underlying lesion by angioplasty with or without stenting. It is for this reason that the outcomes using such approaches are superior.

Although these treatments are available, it would be preferable if thrombosis could be prevented. The use of systemic antithrombotic therapies in vascular access has not been demonstrated to reduce the incidence.^11^,^12^,^21^ Antiplatelets (aspirin, clopidogrel, dipyrimadole) have not shown any benefit and formal anticoagulation with warfarin has shown worse outcomes. Strategies with coating grafts with heparin (Propaten) again have not demonstrated statistically significant benefit in vascular access.^19^ The failure of these approaches further confirms that thrombosis is not due to a single factor but is frequently associated with an underlying lesion and is precipitated by concomitant risk factors.

In summary thrombosis remains the main cause of vascular access failure and although multiple treatments are available, the prevention lies in detecting stenosis before they become critical.
